# Efficacy of collagenase in patients who did and did not have previous hand surgery for Dupuytren's contracture

**DOI:** 10.3109/2000656X.2012.683795

**Published:** 2012-06-07

**Authors:** Chris Bainbridge, Robert A. Gerber, Piotr P. Szczypa, Ted Smith, Harvey Kushner, Brian Cohen, Marie-Pierre Hellio Le Graverand-Gastineau

**Affiliations:** 1 Pulvertaft Hand Clinic, Royal Derby Hospital, Derby, UK; 2 Pflzer Inc Medicines Development Group Groton, CT, USA; 3 Medical Affairs Pfizer Ltd, Tadworth, Surrey, UK; 4 Auxilium Pharmaceuticals, Malvern, PA, USA

**Keywords:** Dupuytren's disease, surgery, recurrence, collagenase, contracture, range of motion

## Abstract

Collagenase *Clostridium histolyticum* (CCH) is a non-surgical, efficacious therapy for Dupuytren's contracture (DC). This study evaluated the efficacy and safety of CCH in patients with previous DC surgery. Data from 12 CCH clinical trials were pooled. At screening, patients provided details about the type/date of previous DC surgery. Reviewers coded descriptions to the Operated Hand, finger, and joint. Of 1082 patients, 422 (39%) had previous DC surgery. For these patients with previous surgery, the CCH treatment was coded on the Operated (*n* = 206) or Non-operated Hand (*n* = 196). End-points included changes in fixed-flexion contracture (FFC) and range of motion (ROM). Adverse events (AEs) were monitored. After treatment with CCH, FFC at metacarpophalangeal joints was reduced by 75% in previously Operated Hands and by 80% for Non-operated Hands (*p* = 0.6). Improvements in ROM were 32° and 32°, respectively (*p* = 0.9). For proximal inter-phalangeal joints, the reductions in FFC for the Operated and Non-operated Hands were 52% and 50%, respectively (*p* = 0.6); improvements in ROM were 24° and 26°, respectively (*p =* 0.3). Some AE rates were significantly higher in the Operated vs Non-operated Hand groups, but were not clinically relevant. There were no between-group significant differences in AE duration (*p* > 0.08). Previous surgery for DC does not affect efficacy or safety of CCH, suggesting CCH is an option in patients with recurring DC. Some AE rates were significantly higher, but not clinically relevant.

## Introduction

Dupuytren's disease is a progressive, fibro-proliferative disorder affecting the palmar fascia whereby early nodular tissue develops intoathick collagen cord.Asthe cordcontracts,flexion deformity of the affected metacarpophalangeal (MP) or proximal inter-phalangeal (PIP) joint ensues. Joint contracture is a common presenting complaint, as affected individuals have difficulty performing a variety of tasks. Many are also embarrassed by the visible deformity [1]. Thereisno cure for Dupuytren's disease and corrective surgery is the current standard of care for Dupuytren's contracture (DC). Surgical approaches involve excision (fasciectomy) ordivision (fasciotomy) ofthe cord in affected finger(s). Although surgery can improve outcomes, recurrence is commonand patients frequently require re-treatment. Surgical re-treatment may be complex and results in higher complication rates compared with primary procedures.

Collagenase *Clostridium histolyticum* (CCH) was recently approved in the US and the EU as the first non-surgical, office-based pharmacotherapy for DC and has proven efficacy in cor-rectingcontractures[2–4].Usingdatafromtheseandotherclinical trials,weevaluated the efficacy and safety of CCH in patients with DC who had undergone previous surgery for the condition.

## Patients and methods

### Study design and patient population

Data from 12 CCH clinical trials conducted globally (Australia, Europe, and US) were pooled. Four studies were randomised, double-blind, placebo-controlled trials; the remaining eight studies were designed as open-label trials. Overall, eligible patients (aged ≥18 years) were in good health but had DC of an MP joint between 20° and 100° and/or a PIP joint between 20° and 80° in ≥1 finger(s) (excluding the thumb). Additionally, results of table-top tests had to be positive, i.e. patients were unable to simultaneously place their affected finger(s) and palm flat on a table. Patients with recurrent disease were eligible to participate if other inclusion/exclusion criteria were met. Further details for some studies have been published [2–4]. All patients provided written informed consent and all studies were conducted under the auspices of the Human Research Ethics Committees at each of the participating centres and in accordance with Good Clinical Practice guidelines [5].

### Treatment/treatment cycles

Before initiating CCH treatment, investigators measured all affected joints and classified contractures into ‘Low-’ and ‘High-severity’ categories. Low-severity contractures were defined as ≤50° MP or ≤40° PIP. High-severity contractures were defined as >50° MP or >40° PIP. Although study designs differed slightly, in general, patients could receive ≤3 CCH (0.58 mg) injections/cord at 30-day intervals. If needed, joints were manipulated the day after an injection in an attempt to rupture the cord. Follow-up visits occurred 1, 7, and 30 days after the injection. A treatment cycle comprised the injection, manipulation, and 30-day follow-up. Each affected cord could undergo a maximum of three treatment cycles. Each patient could receive a maximum of eight treatment cycles; the maximum allowed was three cycles in the double-blind trials and five cycles in the open-label studies. Some patients participated in both a double-blind trial and its open-label extension.

### Assessment of efficacy and safety

For all studies, fixed-flexion angles were measured using finger goniometry at screening and all subsequent visits. The primary end-point was clinical success, defined as a reduction in joint contracture to ≤5° of normal 30 days after the last injection. Secondary efficacy end-points included percentage change from baseline in fixed-flexion contracture (FFC) and change from baseline in range of motion (ROM). In five studies, additional subjective assessments were used. After treatment, patients rated their satisfaction with treatment on a 5-point scale (ranging from Very Satisfied to Very Dissatisfied). Patients were monitored for local and systemic adverse events (AEs), which were assessed for severity and relationship to study treatment and were recorded from first injection to study end.

### Surgical history

**Figure 1:**
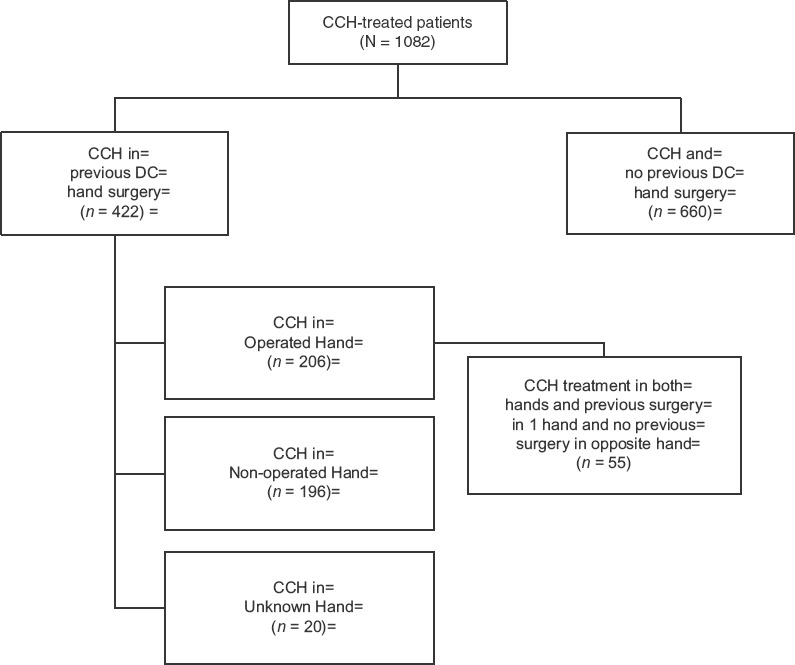
Patient cohorts.

At screening, patients were asked if they had had previous surgery for DC and, if so, to provide details about the type/date of surgery. Two independent reviewers coded all verbatim descriptions to the hand, finger and joint receiving surgery; descriptions were also coded for the type of surgery performed. Although not specifically stated, it was assumed that patients who had >1 surgery provided information for the most recent procedure. All coding was compared at the joint and patient levels. For all joints that received CCH, if the joint was in a hand that received previous surgery, the joint was coded as ‘Operated Hand’, which means that the joint was part of a hand that had previously received surgery. If the CCH-treated joint was in a hand that never received surgery, the joint was coded as ‘Non-operated Hand’. If the Operated Hand was not specified, the CCH-treated joint was coded as ‘Unknown Hand’ (Figure 1).

At the patient level, if the patient had at least one CCH-treated hand that matched the previously Operated Hand, the patient was coded as ‘Operated Hand’. If the Operated Hand was not specified but the patient had CCH treatments on both hands, the patient was also classified as ‘Operated Hand’. If the patient did not receive any CCH treatments to the previously Operated Hand, the patient was classified as ‘Non-operated Hand’. If the previously Operated Hand was not specified and the patient received CCH treatment in one hand, the patient was classified as ‘Unknown Hand’. In addition, a small cohort of patients was identified that had previous surgery in one hand and CCH treatment in both hands.

### Statistical analyses

For continuous data, mean ± standard deviations (SD) were reported unless otherwise specified. For baseline demographic and clinical characteristics, a one-way analysis of variance (ANOVA) was used to test for differences between means; a Fisher's exact test was used for differences in proportions. The primary efficacy analyses were limited to all patients who received previous surgery for DC and were based on comparisons of CCH-treated joints in hands that had been surgically treated vs CCH-treated joints in hands that had not been surgically treated. Differences in the primary end-point (clinical success) were tested using logistic regression with baseline severity, joint type and surgery or surgery to the same hand as predictor variables and all possible interaction terms. Differences in FFC and ROM were tested using repeated-measures, mixed-effects model with joint type, baseline severity and surgery or surgery to the same hand as fixed effects and the patient as the random effect with all possible interaction terms.

For the small cohort of patients that had undergone previous surgery in one hand and received CCH treatment in both hands, patients served as their own controls (matched-pairs cohort). Patients in this cohort could have more than one joint in each of the hands treated with CCH. Differences in clinical success rates for this matched-pairs cohort were analysed using a conditional logistic regression model with the baseline severity as a cova-riate. Changes in FFC and ROM were compared using the mixed-effects model.

All data from patients categorised to the Unknown Hand group were excluded from analysis. An alpha level ≤0.05 was used to determine statistical significance; Bonferroni corrections were not applied. All analyses were performed using SAS Version 9.1 (SAS Institute Inc, Cary, NC).

## Results

### Patient demographics and disposition

Data from 1082 patients who received ≥1 CCH injection were analysed and coded for both patients and joints. Nearly 40% of patients had received previous surgery for DC. Among these, 49% and 46% had CCH treatment on the Operated Hand and Non-operated Hand, respectively. The Operated Hand could not be identified for 5% of patients (Unknown Hand) (Table I). For 30% of patients, the type of surgery was recorded: 55% excisions (i.e. fasciectomy, aponeurectomy, capsulectomy), 41% divisions (i.e. release, fasciotomy, aponeurotomy) and 5% amputations. Median time from the most recent surgery to study entry was 5 years; 30% of surgeries were performed within 3 years of the patient's first study visit; 25% were performed >9 years before the patient's first study visit. For baseline characteristics, there were some statistically significant differences between patients who had and had not undergone previous DC surgery. Patients in the Previous Surgery group had more severe disease on a number of indices (Table II).

**Table I:** CCH treatment in patients with or without previous surgery for DC by patients and joints.

	Previous Surgery Groups				
		
	CCH in Operated Hand	CCH in Non-operated Hand	CCH in Unknown Hand	CCH in Surgery Total	CCH and No Surgery
Patients, *n*	206	196	20	422	660
Treated joints, *n*	317	364	42	723	1055
MP, *n* (%)*	131 (41)	214 (59)	21 (50)	366 (51)	669 (63)
PIP, *n* (%)**	186 (59)	150 (41)	21 (50)	357 (49)	386 (37)
Injections/joint					
Mean ± SD	1.5 ± 0.8	1.6 ± 0.8	1.4 ± 0.7	1.5 ± 0.8	1.5 ± 0.7
Median	1.0	1.0	1.0	1.0	1.0

* *p* < 0.001 for MP:PIP ratio for Previous Surgery vs No Previous Surgery.

** *p* < 0.001 for MP:PIP ratio for CCH treatment on the Operated Hand vs CCH treatment on the Non-operated Hand. CCH, collagenase *Clostridium histolyticum*; DC, Dupuytren's contracture; MP, metacarpophalangeal; PIP, proximal inter-phalangeal; SD, standard deviation.

**Table II:** Baseline demographics and clinical characteristics for patients treated with CCH with or without previous surgery for DC.

	Previous Surgery Groups				*p*-value	
		
Characteristic	CCH and No Surgery (*n* = 660)	CCH in Operated Hand (*n* = 206)	CCH in Non-operated Hand (*n* = 196)	CCH in Unknown Hand (*n* = 20)	No Surgery vs Previous Surgery	CCH in Operated Hand vs CCH in Non-operated Hand
Mean ± SD age, years	63 ± 9	63 ± 10	64 ± 10	58 ± 8	0.77	0.63
Male gender, *n* (%)	547 (83)	172 (84)	165 (84)	18 (90)	0.62	0.89
BMI category, *n* (%)					0.73	0.22
Normal (<25 kg/m*m)	250 (38)	71 (35)	76 (39)	9 (45)		
Overweight (25-29 kg/m*m)	285 (43)	102 (50)	80 (41)	10 (50)		
Obese (>30 kg/m*m)	123 (19)	33 (16)	39 (20)	1 (5)		
Mean ± SD age at diagnosis, years	56 ± 12	48 ± 13	51 ± 12	45 ± 11	< 0.001	0.03
Mean ± SD duration of DD, years	7.6 ± 7.6	14.8 ± 10.2	12.6 ± 8.9	12.4 ± 8.0	< 0.001	0.02
Mean ± SD joints affected, *n*	2.6 ±1.9	3.5 ± 2.3	3.0 ± 2.0	2.9 ±1.5	< 0.001	0.02
Mean ± SD TCI^a^	127 ± 106 [*n* = 589]	180 ± 122 [*n* = 181]	149 ± 109 [*n* = 174]	137 ± 76 [*n* = 18]	< 0.001	0.01
Hands affected, *n* (%)					0.01	< 0.001
One	385 (58)	83 (40)	119 (61)	8 (40)		
Both	192 (29)	98 (48)	53 (27)	9 (45)		
Missing information	83 (13)	25 (12)	24 (12)	3 (15)		
Family history of DD, *n* (%)	255 (39)	108 (52)	87 (45)	13 (65)	< 0.001	0.12
Other medical history, *n* (%)						
Peyronie's disease	17 (3)	7 (3)	10 (5)	0 (0)	0.21	0.46
Ledderhose's disease	21 (3)	12 (6)	10 (5)	0 (0)	0.11	0.83
Physician-rated severity, *n* (%)					0.01	0.01
Mild	139 (21)	30 (10)	40 (20)	3 (15)		
Moderate	319 (48)	101 (49)	85 (43)	10 (50)		
Severe	120 (18)	59 (29)	47 (24)	4 (20)		
Missing information	82 (12)	26 (13)	24 (12)	3 (15)		

a Sum of the fixed-flexion contracture of all 16 joints (excluding DIP joints and thumb) at screening. If a joint had a fixed-flexion contracture not caused by a Dupuytren's cord, the fixed-flexion contracture was set to 0. BMI, body mass index; CCH, collagenase *Clostridium histolyticum*; DC, Dupuytren's contracture; DD, Dupuytren's disease; SD, standard deviation; TCI, total contracture index.

Within the Previous Surgery cohort of patients for which the Operated Hand could be identified (*n* = 402), there were significant differences between those who received CCH on the same hand as the surgery (*n* = 206) and patients who received CCH on the opposite hand (*n* = 196). Overall, patients in the Operated Hand group had greater disease severity than did patients in the Non-operated Hand group. Nevertheless, the Surgery cohorts were more similar to each other than to the No Surgery cohort. About 60% of patients in the Operated Hand group had previous surgery on both hands and/or had CCH treatment on both hands.

### Efficacy

**Figure 2:**
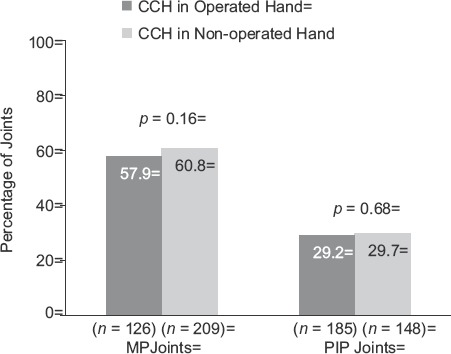
Reduction in contracture to ≤5° at 30 days after the last CCH injection among patients with previous surgery in CCH-treated joints in the previously Operated Hand vs CCH-treated joints in the Non-operated Hand. CCH, collagenase *Clostridium histolyticum*; MP, metacarpophalangeal; PIP, proximal inter-phalangeal.

**Figure 3:**
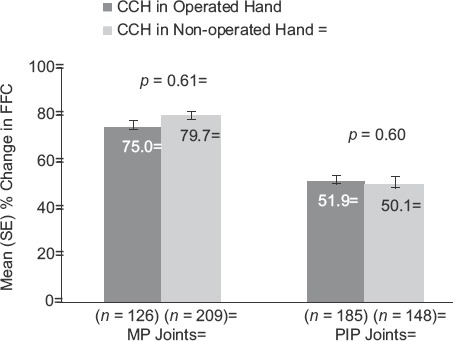
Percentage change in FFC among patients with previous surgery in CCH-treated joints in the previously Operated vs CCH-treated joints in the Non-operated Hand. CCH, collagenase *Clostridium histolyticum*; FFC, fixed-flexion contracture; MP, metacarpophalan-geal; PIP, proximal inter-phalangeal.

**Figure 4:**
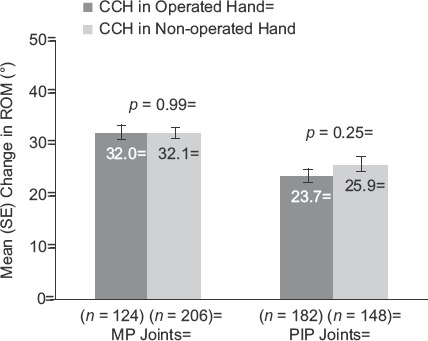
Change in ROM among patients with previous surgery in CCH-treated joints in the previously Operated vs CCH-treated joints in the Non-operated Hand. CCH, collagenase *Clostridium histolyticum*; MP, metacarpophalangeal; PIP, proximal inter-phalangeal; ROM, range of motion.

For the primary end-point of clinical success, there were no statistically significant between-group differences in the percentage of patients (Operated vs Non-operated Hand) who showed a reduction in contracture to ≤5° 30 days after the last CCH injection for MP (57.9% and 60.8%, respectively; *p* = 0.16) or PIP joints (29.2% and 29.7%, respectively;p = 0.68; Figure 2). After CCH treatment, reductions in FFC were 75% (Operated Hand) and 79.7% (Non-operated Hand; *p* = 0.61) for MP joints and 51.9% (Operated Hand) and 50.1% (Non-operated Hand; *p* = 0.60) for PIP joints (Figure 3). Improvements in ROM were 32.0° and 32.1°, respectively, for MP joints (*p* = 0.99), and 23.7° and 25.9° for PIP joints (*p* = 0.25; Figure 4). There was no difference in the proportion of patients who were satisfied with treatment in the Operated Hand (81%) and Non-operated Hand groups (88%; *p* = 0.23).

Statistical comparisons between the Surgery sub-groups and the No Surgery group are confounded by factors described above and shown in Table II. However, clinical success rates for MP and PIP joints were significantly higher among patients who did not have previous hand surgery vs all patients who did (*p* < 0.001). Similarly, changes in FFC and ROM were significantly higher in the No Surgery group than the Surgery group (*p* = 0.009).

**Figure 5:**
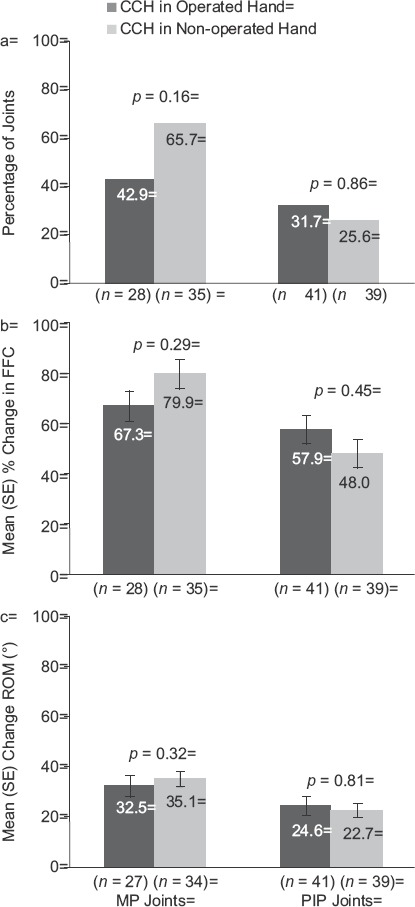
Results for the matched-pairs cohort: reduction in contracture to ≤5° at 30 days after the last CCH injection (*a*), change in FFC (*b*), change in ROM (c). CCH, collagenase *Clostridium histolyticum*; FFC, fixed-flexion contracture; MP, metacarpophalangeal; PIP, proximal inter-phalangeal; ROM, range of motion.

In the matched-pairs cohort (*n* = 55) that had undergone surgery in one hand and received CCH treatment in both hands, previous surgery and treatment efficacy could be evaluated more directly, as patients served as their own controls. Conditional logistic regression for clinical success and repeated-measures analyses for changes in FFC and ROM did not show any statistically significant differences between hands or between MP or PIP joints (Figure 5).

### Safety

Overall, 97% of patients who received CCH injections reported ≥1 treatment-related AEs (Table III). The incidence of three AEs (injection site haemorrhage, blood blister, axillary pain) was significantly higher in the Previous Surgery vs No Surgery groups (*p* < 0.05). Rates for six AEs (oedema peripheral, contusion, ecchymosis, pain in extremity, tenderness, lymph-adenopathy) were significantly higher in the Operated Hand vs Non-operated Hand groups (*p* < 0.05). There was complete overlap in AEs experienced by patients in the two Surgery subgroups; none was unique to either group. Most treatment-related AEs were mild or moderate and resolved without intervention (median 8–10 days).

**Table III:** Treatment-related adverse events occurring in ≥5% of patients treated with CCH with or without Previous Surgery for DC.

		Previous Surgery Groups, *n* (%)			*p*-value*	
			
Characteristic	CCH and No Surgery (*n* = 660)	CCH in Operated Hand (*n* = 206)	CCH in Non-operated Hand (*n* = 196)	CCH in Unknown Hand (*n* = 20)	No Surgery vs Previous Surgery	CCH in Operated Hand vs CCH in Non-operated Hand
≥1 treatment-related adverse event	641 (97)	204 (99)	187 (95)	19 (95)	–	–
Oedema peripheral†	504 (76)	172 (84)	148 (76)	14 (70)	0.30	0.05
Contusion	353 (54)	131 (64)	95 (49)	10 (50)	0.45	0.01
Injection site pain	272 (41)	74 (36)	87 (44)	7 (35)	0.66	0.09
Pain in extremity	228 (35)	100 (48)	58 (30)	9 (45)	0.11	< 0.001
Injection site haemorrhage	211 (32)	73 (35)	81 (41)	6 (30)	0.05	0.26
Tenderness	184 (28)	70 (34)	47 (24)	9 (45)	0.49	0.03
Injection site swelling	166 (25)	41 (20)	54 (28)	3 (15)	0.50	0.08
Ecchymosis	125 (19)	45 (22)	24 (12)	0 (0)	0.29	0.01
Pruritus	82 (12)	31 (15)	20 (10)	3 (15)	0.85	0.18
Skin laceration	78 (12)	24 (12)	16 (8)	3 (15)	0.43	0.32
Lymphadenopathy	65 (10)	36 (18)	17 (9)	2 (10)	0.11	0.01
Blood blister	46 (7)	30 (15)	19 (10)	2 (10)	0.01	0.17
Axillary pain	32 (5)	23 (11)	16 (8)	1 (5)	0.01	0.32
Haematoma	31 (5)	11 (5)	12 (6)	2 (10)	0.40	0.83

* Based on Fisher's exact test.

† Oedema of the treated extremity, not diffuse oedema in all extremities. CCH, collagenase Clostridium histolyticum; DC, Dupuytren's contracture.

In all, nine patients experienced 10 serious AEs deemed possibly or probably related to treatment. In the No Surgery group, this included a recurrence of complex regional pain syndrome (CRPS), one tendon rupture, a boutonniere deformity and, in one patient, a case of sensory disturbance and thickening of a Dupuytren's cord. In the Previous Surgery/Operated Hand group, there were two tendon ruptures, lower extremity deep vein thrombosis and tendonitis; in the Non-operated Hand group, one ligament injury was reported.

### Discussion

Dupuytren's disease is a chronic and progressive condition with no cure. While surgery can reduce contracture and restore hand function, these improvements are generally not permanent. Almost inevitably, recurrence and/or extension will occur and re-treatment will be indicated. Thus, it is important to monitor the long-term efficacy and safety of repeated—oftentimes multiple—surgeries for recurrent contractures. Recently, CCH was approved as the first non-surgical, minimally invasive pharmacotherapy for DC with a palpable cord. Using data from 12 CCH clinical trials, we retrospectively evaluated the efficacy and safety of CCH in patients who had and had not undergone previous surgery for the condition.

Using strictly defined inclusion criteria, 40% of the 1082 patients were identified as having had previous DC surgery. The proportions of patients treated with CCH after surgery in the Operated Hand vs the Non-operated Hand group were evenly distributed, as were the number of MP and PIP joints treated. There were no differences in the number of CCH injections in any group. Importantly, when comparing the Previous Surgery groups with the No Surgery group on efficacy measures, it must be kept in context that, owing to significant differences in baseline characteristics, particularly those related to disease severity, having a previous surgery is probably confounded with disease severity.

For efficacy measures, including reduction in contracture to ≤5° and improvements in FFC and ROM, there were no statistically significant differences between the Previous Surgery sub-groups. Recently, the clinically important difference (CID) for ROM was determined to be 13.5°. That is, an improvement in ROM >13.5° is associated with clinically relevant changes from the patient and physician perspectives [6]. In the current analysis, both surgery sub-groups showed clinically significant improvements from baseline for both MP and PIP joints. Moreover, the magnitude of the differences in ROM between the groups (i.e. <2.2°) was substantially less than the CID, indicating no clinically meaningful differences on this end-point. There was no difference in patient satisfaction rates and, with few exceptions, there were no significant differences in the tolerability profiles between the Previous Surgery sub-groups. There was, however, a trend for higher AE rates in the Operated Hand vs Non-operated Hand groups.

Our findings are largely consistent with earlier published reports, particularly in the context of the handful of studies directly comparing the results of surgery for primary vs recurrent contractures. Some of these retrospective evaluations of outcomes after fasciectomy examined clinical and functional improvements and others focused on postoperative complications. A small number evaluated both. For efficacy, most studies evaluated improvements in contracture and results varied [7–9]. Coert et al. [8] reported that, for MP joints, postoperative gains in FFC were 94% for primary and 91% for recurrent surgeries (*p* = 0.013). For PIP joints, FFC gains were 72% and 68%, respectively (*p* = 0.118). In another study [10], 78% of primary and 69% of recurrent surgeries of MP joints produced correction of contracture to 100% (not significant). For PIP joints, 32% of primary and 12% of secondary surgeries showed 100% correction (p < 0.05). Our results were consistent with these studies for MP but not PIP joints. For MP joints, changes in FFC were greater, albeit not statistically, in the Non-operated Hand vs Operated Hand group. For PIP joints, our results showed greater, again not statistically, FFC improvements in the Operated Hand vs Non-operated Hand group. Belusa et al. [7] also reported greater improvements in FFC after recurrent vs primary surgeries for PIP joints of the ring (62% vs 50%) and small fingers (46% vs 42%, respectively).

Treatment-related AEs occurring in ≤5% of patients were, with few exceptions, higher in the Operated Hand vs Non-operated Hand group; however, for all AEs that were significantly different between the two groups, rates were higher in the Operated Hand vs Non-operated Hand group. There were more serious AEs in the Operated Hand vs Non-operated Hand group. To put these findings in context of the current literature, several studies have reported on overall complication rates [11–13], nerve injuries [8,12–15], artery injuries [14,15], and stiffness [15] after fasciectomy. Ebskov et al. [12] also reported on the occurrence of infection, skin necrosis, and haematoma. Two studies reported no notable differences in overall complication rates between primary and recurrent surgery groups [11,12]; Hogemann et al. [13] reported a lower complication rate in the primary vs recurrent groups (11% vs 38%). Three studies reported lower rates of perioperative nerve and artery injuries in primary vs recurrent groups [8,14,15]. Ebskov et al. [12] noted a higher rate of nerve injury in the recurrent vs primary group (10% vs 4%) and Coert et al. [8] reported a higher incidence of CRPS among primary vs recurrent surgeries.

The findings should be considered in the light of some limitations. Data were pooled from 12 clinical trials that differed in overall design (e.g. double-blind, placebo-controlled, and open-label) and the number of joints that could be treated. However, all joints could receive up to three CCH treatment cycles and efficacy and safety evaluations did not differ among trials. Patient surgical history was based on self-report and not confirmed with chart reviews. Although the two evaluators reached consensus on their ratings, important information was not provided, especially with regard to the fingers and joints receiving surgery, types of surgeries performed, and the time when they occurred. Also, surgery on the same hand may or may not indicate recurrence of disease in the treated joint. The matched-pairs analysis was based on a small cohort of 55 subjects but, to our knowledge, it is the largest such paired analysis performed to date.

The literature on Dupuytren's disease is rife with studies examining the outcomes and complications of surgery for DC and the body of evidence for the efficacy of CCH in correcting DC is growing. Without a cure, however, contractures will recur and re-treatment will be necessary. Surgical re-treatment can be complex, as internal scar tissue can integrate with the Dupuytren's cord, arteries, nerves, and tendons. Moreover, surgical re-treatment is generally associated with higher complication rates vs the primary procedure. Thus, minimally invasive techniques such as CCH treatment may become a preferred approach to DC recurrence for physicians and patients alike. For physicians, it is an easier and less risky procedure. For patients, it provides an alternative strategy for those who are reluctant to undergo a second (or third) surgery.

Taken together, our preliminary data suggest that CCH, as a non-surgical treatment, is efficacious and well tolerated in patients who have or have not undergone previous surgery for DC. Future prospective studies are warranted to better evaluate the longer-term effects of repeated treatments for this debilitating disease.
